# Supplementary material for: Causal Relationship between Aging and Anorexia Nervosa: A White-Matter-Microstructure-Mediated Mendelian Randomization Analysis
Source: Biomedicines. 2024 Aug 16;12(8):1874. doi: 10.3390/biomedicines12081874 (PMC11351342; doi:10.3390/biomedicines12081874)
Supplement: Supplementary file 1 [file biomedicines-12-01874-s001.zip › Supplementary Figure/Figure S4/Singe SNP plot of MR analysis for TL to ubm-b-1663.pdf]

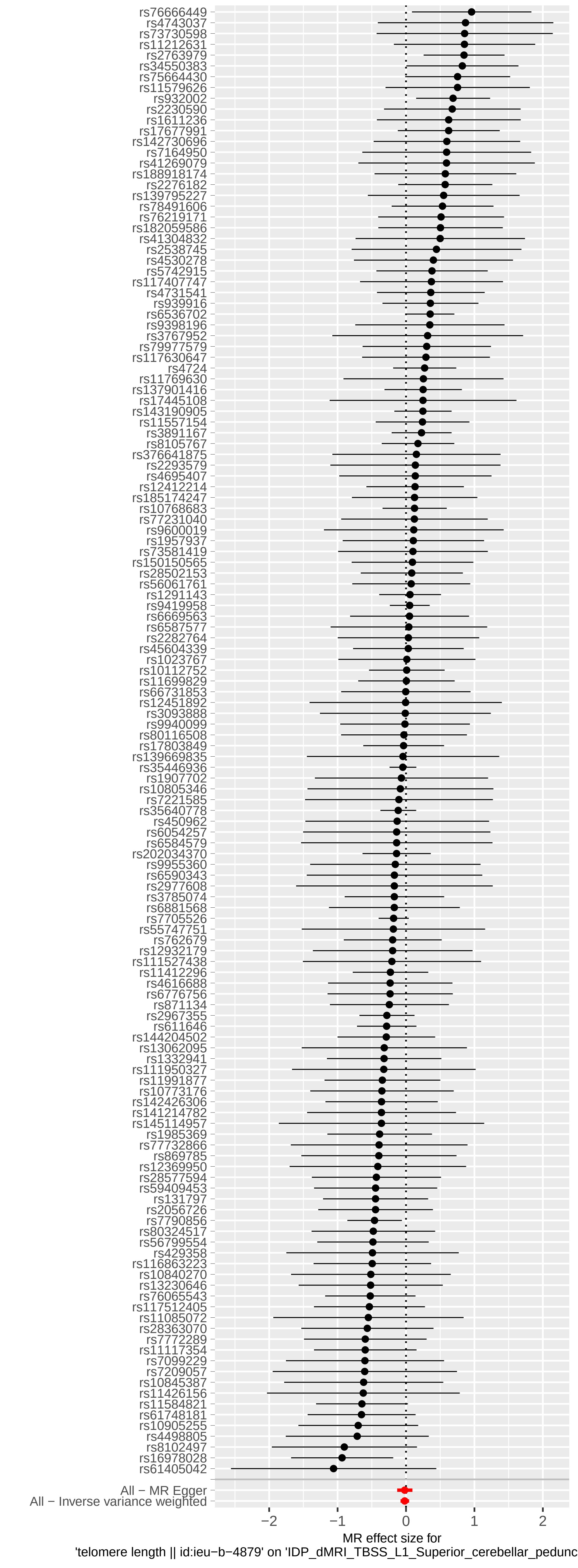
rs76666449
rs4743037
rs73730598
rs11212631
rs2763979
rs34550383
rs75664430
rs11579626
rs932002
rs2230590
rs1611236
rs17677991
rs142730696
rs7164950
rs41269079
rs188918174
rs2276182
rs139795227
rs78491606
rs76219171
rs182059586
rs41304832
rs2538745
rs4530278
rs5742915
rs117407747
rs4731541
rs939916
rs6536702
rs9398196
rs3767952
rs79977579
rs117630647
rs4724
rs11769630
rs137901416
rs17445108
rs143190905
rs11557154
rs3891167
rs8105767
rs376641875
rs2293579
rs4695407
rs12412214
rs185174247
rs10768683
rs77231040
rs9600019
rs1957937
rs73581419
rs150150565
rs28502153
rs56061761
rs1291143
rs9419958
rs6669563
rs6587577
rs2282764
rs45604339
rs1023767
rs10112752
rs11699829
rs66731853
rs12451892
rs3093888
rs9940099
rs80116508
rs17803849
rs139669835
rs35446936
rs1907702
rs10805346
rs7221585
rs35640778
rs450962
rs6054257
rs6584579
rs202034370
rs9955360
rs6590343
rs2977608
rs3785074
rs6881568
rs7705526
rs55747751
rs762679
rs12932179
rs111527438
rs11412296
rs4616688
rs6776756
rs871134
rs2967355
rs611646
rs144204502
rs13062095
rs1332941
rs111950327
rs11991877
rs10773176
rs142426306
rs141214782
rs145114957
rs1985369
rs77732866
rs869785
rs12369950
rs28577594
rs59409453
rs131797
rs2056726
rs7790856
rs80324517
rs56799554
rs429358
rs116863223
rs10840270
rs13230646
rs76065543
rs117512405
rs11085072
rs28363070
rs7772289
rs11117354
rs7099229
rs7209057
rs10845387
rs11426156
rs11584821
rs61748181
rs10905255
rs4498805
rs8102497
rs16978028
rs61405042
All − MR Egger
All − Inverse variance weighted
−2
−1
0
1
2
MR effect size for
'telomere length || id:ieu−b−4879' on 'IDP_dMRI_TBSS_L1_Superior_cerebellar_pedunc
